# Automotive Radar in a UAV to Assess Earth Surface Processes and Land Responses

**DOI:** 10.3390/s20164463

**Published:** 2020-08-10

**Authors:** Christoph Weber, Johannes von Eichel-Streiber, Jesús Rodrigo-Comino, Jens Altenburg, Thomas Udelhoven

**Affiliations:** 1 Engineering, Computer Science and Economics, TH Bingen University of Applied Sciences, 55411 Bingen am Rhein, Germany; j.altenburg@th-bingen.de; 2 Institute for Innovation, Transfer and Consulting, 55411 Bingen am Rhein, Germany; johannes_von_eichel@gmx.de; 3 Erosion and Degradation Research Group, Department of Geography, University of Valencia, 46010 Valencia, Spain; jesus.rodrigo@uv.es; 4 Physical Geography, University of Trier, 54286 Trier, Germany; 5 Environmental Remote Sensing & Geoinformatics Department, University of Trier, 54286 Trier, Germany; udelhove@uni-trier.de

**Keywords:** Radar, UAV, 77 GHz, ARS-408, ARS-404

## Abstract

The use of unmanned aerial vehicles (UAVs) in earth science research has drastically increased during the last decade. The reason being innumerable advantages to detecting and monitoring various environmental processes before and after certain events such as rain, wind, flood, etc. or to assess the current status of specific landforms such as gullies, rills, or ravines. The UAV equipped sensors are a key part to success. Besides commonly used sensors such as cameras, radar sensors are another possibility. They are less known for this application, but already well established in research. A vast number of research projects use professional radars, but they are expensive and difficult to handle. Therefore, the use of low-cost radar sensors is becoming more relevant. In this article, to make the usage of radar simpler and more efficient, we developed with automotive radar technology. We introduce basic radar techniques and present two radar sensors with their specifications. To record the radar data, we developed a system with an integrated camera and sensors. The weight of the whole system is about 315 g for the small radar and 450 g for the large one. The whole system was integrated into a UAV and test flights were performed. After that, several flights were carried out, to verify the system with both radar sensors. Thereby, the records provide an insight into the radar data. We demonstrated that the recording system works and the radar sensors are suitable for the usage in a UAV and future earth science research because of its autonomy, precision, and lightweight.

## 1. Introduction

During the last years, the development of unmanned aerial vehicles (UAVs) has rapidly increased to assess different environmental issues related to earth surface processes and landform responses [1,2]. One of the most important reasons for this is the miniaturization of electronic components and sensors, making their installation and use more accurate and affordable [3,4]. After the improvement reached an acceptable level, which allows building UAVs with high performances sensors at a low price, everyone is practically able to fly and operate UAVs without knowledge of the technical complexity inside the devices [5]. The acceptance and demand of the customers let grown the UAV to a mass-market product [4]. Besides the private customers, the UAV is already accepted in the research as well. A vast number of research projects and technical applications use UAVs to investigate, e.g., landslide and soil erosion monitoring [6,7,8,9], landmine detection [10,11], vegetation and land cover monitoring [12,13], or disaster zone surveillance [14,15].

Next to the actual UAV, the equipped sensor can be considered as the most important part of the system. Most UAVs are characterized by a basic commercial camera, but the use of special sensors increases [16]. Yao et al. [17] exposed an overview about different sensors such as multispectral cameras, hyperspectral sensors, infrared sensors, and Lidar sensors (Light detection and ranging), which show multiple use cases and applications. Among these sensors, radar sensors (Radio detection and ranging) can show new applications and research ways related to earth surface processes and land responses. The basics of radar technology are described in the standard literature [18,19,20,21]. Most radar sensors are short- or mid-range sensors, only measuring the distance, need a complex evaluation of raw data or do not match UAVs requirements and thereby useless for non-radar expert researchers (e.g., radar products from [22,23]). Unfortunately, high investment is necessary to get a radar with specifications that allows using it in high-accurate research. In addition, the weight of the equipment is considered as a problem. According to [24,25,26,27], some projects utilize professional radar sensors. In addition to these, there are several projects aiming at developing low-cost radar sensors usable for a variety of applications [28,29,30]. The combination however, a professional low-cost radar sensor is a gap. In order to fill this gap, the use of automotive grade radar sensors could be a solution.

Nowadays, radar sensors are widely used in automotive applications such as a collision avoidance system (CAS), lane change assistance (LCA), adaptive cruise control (ACC), or an emergency brake system (EBS) [31,32]. The idea of using radar as a driver assistance system was first presented in 1955 in the German magazine “Auto, Motor & Sport”. It would be several decades before the first series production. In the seventies, a major research project was founded with the goal to develop a CAS. Thereby, the radar technology made significant progress and became more popular but a series production was not possible because of technical conditions at this time [31]. The first automotive application using radar technology was an ACC from Mercedes-Benz in 1999 [33]. It included the CAS resulting from the research project founded in the seventies.

To push the radar technology in UAVs to a new level and close the gap between professional and low-cost radar sensors, we used automotive radar technology. The main purpose of this research was to present two automotive grade radar sensors and their usability in scientific research related to earth surface processes and landforms. Even if these sensors do not provide imaging data such as classical remote sensing synthetic-aperture radar applications, the efficient development of this new system would allow improving the detection of gullies and rills [34,35,36,37], forecasting of landslides, flash floods [38,39], river evolution [40,41], and aeolian processes [42,43]. Furthermore, the impacts on land-use changes [44,45,46] due to the interaction between the microwave signal and different microwave absorbing materials in the soil can be evaluated. These devices would be suitable to be combined with in situ experiments or monitoring [47] and with optical data. The main use of the system is assessing the above-mentioned processes before or after specific events such as rain, wind, floods, etc., or analyzing the status of specific landforms. Further, we checked their usability in combination with a UAV. To achieve this goal: (i) two radar sensors were presented with different specifications; (ii) a system was developed to record the radar data; (iii) additional sensors were added; (iv) the whole recording system was integrated into a UAV; (v) measurements were taken to validate the recording system; and, (vi) the results were discussed.

## 2. Materials and Methods

### 2.1. Automotive Radar Technology

It is well known that Radar is based on the spread of electromagnetic waves. The frequency ranges from 3–30 GHz (centimeter waves) to 30–300 GHz (millimeter waves). There are two commonly used frequencies in the automotive sector, one for short-range applications with 24 GHz and the other one for long-range with 77 GHz. Both have different performances in terms of resolution, due to smaller wavelength in the latter case.

**Figure 1:**
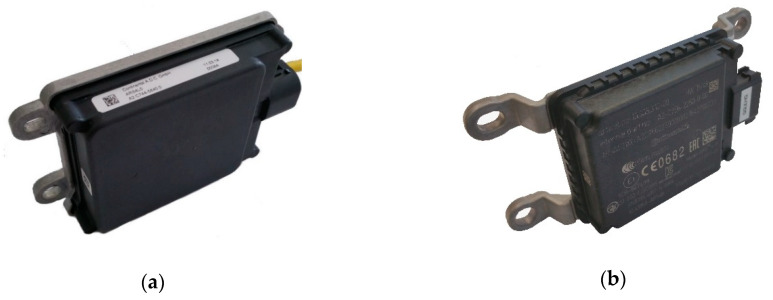
Radar sensors ARS-40X showing. On the right side of the sensors, are the CAN interface connector. (**a**) ARS-408 and (**b**) ARS-404.

For detecting different landforms and assess surface processes, we considered the use of the radar sensors ARS-408 and ARS-404 (Advanced Radar Sensor) from Continental [48] (Figure 1). Besides Continental, other automotive supplier such as Bosch, Hella or TRW produce radar sensors for automotive applications as well. Further, companies such as Sick and Innosent offer radar sensors with focus on industrial applications. From the wide range Continental is the unique enterprise that meets our desired requirements to achieve our goals: (i) low price; (ii) low weight and power consumption; (iii) high robustness; (iv) small size; (v) high range option; and (vi) suitable and easily usable interface.

The ARS-400 radar sensor series originally comes from the automotive industry. These radar sensors use to be built in the front bumper of cars and trucks to observe the area in front of the vehicle. Both are long-range sensors and use 77 GHz with a wavelength of about 3.9 mm. The sensors have different sizes and therefore, different performances whereby the ARS-408 is the larger one with higher performance. Due to a special software adaptation from the subsidiary Continental Engineering Services (CES), the radar sensor can be used for industrial purposes such as area monitoring or collision avoidance for large construction vehicles. Data can be exchanged via the Controller Area Network (CAN) interface. The larger ARS-408 is on the left-hand side (Figure 1a) and the smaller ARS-404 on the right-hand side (Figure 1b). On the right side of the sensors are the CAN and power connector situated.

**Figure 2:**
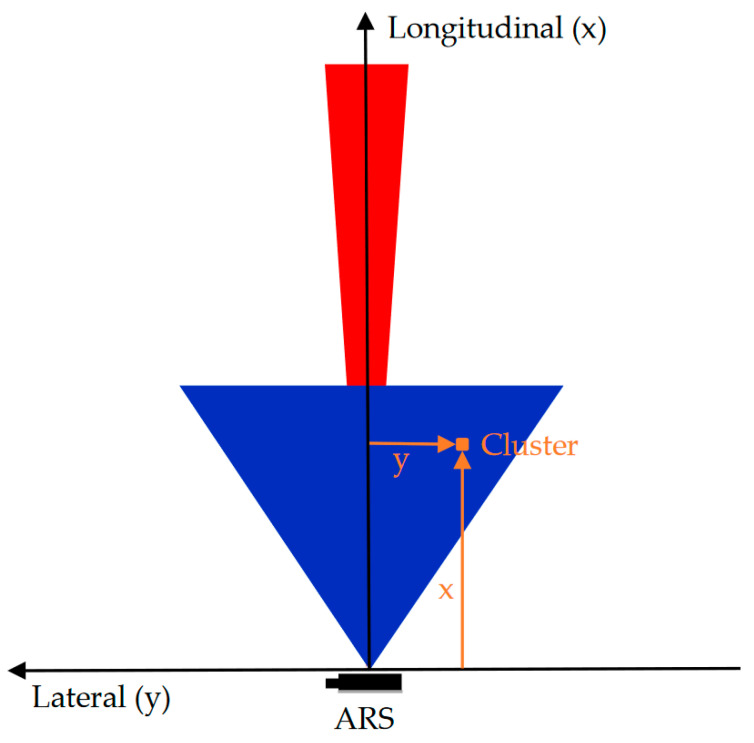
Based on R. Liebske [49] the coordinate system from the radar sensor with one cluster data is described in the longitudinal and lateral axis. In the blue color, the near-field view is represented and in the red, the far-field.

The ARS-400 series has a real aperture and uses the frequency modulated continuous wave (FMCW) process. It has a phased array antenna and the measurement is performed in two dimensions. The detected reflections of the radar are issued as clusters via the CAN interface. Relevant data obtained from a cluster are longitudinal and lateral distances as well as the radar cross-section (RCS) values which represent the reflected power. Contrary to other radar sensors, it is not necessary to start the evaluation of the data at a raw level. The radar output is pre-processed, which means that the process of the raw data and filtering evaluation is already done. Figure 2 shows the coordinate system of the sensor with a cluster and the naming of the axis. Extraordinary for the sensors is that they have two scan areas. Each sensor has two antenna field setups, one for the near-field and one for the far-field measurements. Both fields have naturally different tasks and specifications. The near field is characterized by a short-range and a large opening angle. Therefore, it is used in the automotive field for slow situations such as driving in a city. On the other hand, the far-field is characterized by a long-range and a small opening angle for fast and straight driving modes. Figure 2 shows the field of view of the ARS-408. In the blue color, the near-field is represented and in the red one, the far-field.

To compare the performance of the two sensors, we present Table 1, which shows the most important specifications.

Though the sensors look different, the mounting is the same. This has the advantage that only one bracket is necessary to fit the system for both sensors. This is the reason why the small ARS-404 has a large size in comparison to other specifications such as the weight. However, because of a lower weight, the ARS-404 is more appropriate for the application in a UAV. In contrast to the ARS-408, the performance can be considered something more limited. To compare both sensors, a recording system was developed as follows.

### 2.2. Recording System

To save the data from the radar sensor, it is necessary to connect it to the CAN interface of the sensor. The company, CES, already has a product for the purpose called Radar PLC. It is extensive but not suitable for this application because of its size and weight. Therefore, we used a Raspberry Pi 3B (Rpi) with a CAN shield CanBerryDual V2.1 (sg-electronic-systems.com; SG Electronic Systems SRLS, Via Sicilia 21, 20024 Garbagnate Milanese (Mi), Italy; €40.00). This recording system is lightweight and easy to be integrated into a UAV system. Furthermore, it is simple to expand with several sensors because of the interfaces. We used the operation system (OS) “Raspbian Buster Lite” to save resources. In comparison to the common OS, it has fewer features such as no desktop or extra software tools. The existing open-source software for the RPi allows easy access to the CAN messages. We consider that a better way is to pre-process the CAN messages. To achieve this goal, a software program written in the programming language C was developed. It receives the CAN messages, decodes them to real values and records the resulting data in a file to the SD card.

**Figure 3:**
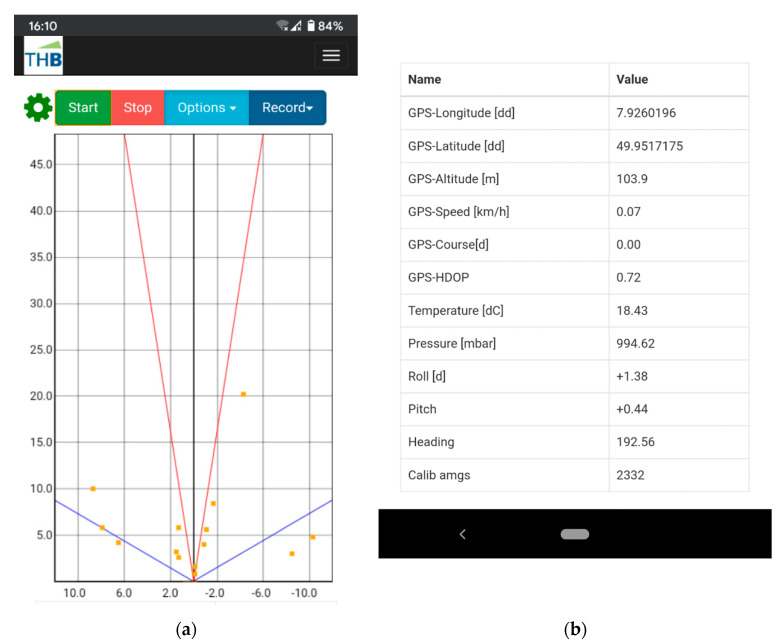
Screenshots obtained from a smartphone with the website aiming to show the live view of the radar data and a table of sensor data. (**a**) Some clusters are shown as orange points in the coordinate system. The buttons on the upper side are used to control the system. (**b**) By scrolling down the website, a table shows the most important sensor data.

Additionally, the CAN shield has a real-time clock (RTC) to get the exact time without an internet connection for a correct log retrace. To control the system, a website was developed as a human-machine interface (HMI). The website has a live view of monitoring the current radar data (see Figure 3a). Furthermore, it allows to start and stop the recording of the radar data and finally, download the records. For that, the software which pre-processes the CAN messages contains a webserver to exchange the data with the website. To reach the website, a device with Wi-Fi is necessary such as smartphone, laptop or tablet to connect to the RPi Wi-Fi. The website can be opened with a normal browser like Chrome or Firefox.

To evaluate the radar data, it is useful to have other sensor data such as position and angle of the radar sensor. Even if the UAV provides this data, the synchronicity with the radar data is not guaranteed. To achieve this goal, a global navigation satellite system (GNSS) and an inertial measurement unit (IMU) are necessary. A second shield is used to expanse the system. For this purpose, we tested two common shields (Table 2). Both shields have a GNSS, IMU, and pressure and temperature sensor. Because of the RPi interfaces, only one GNSS sensor can be applied at a time.

The specifications of the GPS-IMU v3 GNSS sensor are better than the one of the second shield from GlobalTop. It can receive multiple GNSS and has a 10 Hz fix rate. The GlobalTop can only receive GPS signals and has a 5 Hz fix rate. Both sensors were used with an external antenna Taoglas AGGP.25F for a higher reception quality and an independent mounting position of the sensor. The GNSS sensor is connected via a universal asynchronous receiver transmitter (UART) interface to the RPi. The data is coded using the standard National Marine Electronics Association (NMEA) format and can be directly saved to the SD card or pre-processed. We decided to expand the program and pre-process the GNSS data to save only the needed data: Longitudinal and lateral position, altitude, speed, course, and horizontal dilution of precision (HDOP).

The IMU sensors of both shields have 9 degrees of freedom (DOF), which corresponds to the three integrated sensors: accelerometer, magnetometer and gyroscope. All these single sensors measure in three directions X, Y and Z (3 sensors × 3 directions = 9-DOF). The result of a read process is 9 numbers refer to the 9-DOF. The BNO055 has an integrated sensor fusion, thereby the Euler angles can read directly. This is an advantage in comparison to the other IMU that needs a post-process. Both IMU sensors are connected with an Inter-Integrated Circuit (I^2^C) interface. The BNO055 uses a clock stretching method on the I^2^C interface that is not provided by RPi hardware. Therefore, it was changed to a software interface what causes a higher CPU load. The communication is complex and therefore, it is recommended to use an official library of the manufacturer. In this case, it is still necessary to program the communication interface for the library and program a proper sensor set up with the library functions. After that, the values of the IMU can be read and saved to the SD card.

The pressure and temperature sensors are connected with the I^2^C interface as well. The GPS-IMU v3 uses the Bosch BMP280 and on new shields BMP388. Same as for the IMU, the official library is used. For the MS5637 a library from The BlackBoxCamera is available. After communication is established, the two values can be read and saved. After the implementation of the sensors, a complete set of sensor values is available. These recorded sets are saved in a file. For the radar data, an extra file is saved. Further, the most important sensor values are listed on the website shown in Figure 3b. The data of the radar are difficult to interpret because they just point clouds. Therefore, a camera was used as a reference view. The RPi has a camera serial interface and is used with the Raspberry Camera V2. The HMI was extended to start and stop the camera picture recording. The pictures are stored on the SD card to download them.

### 2.3. Integration in the UAV

To use the complete measurement system in a UAV, weight is one of the most important points. In Table 3 the weight of the components is presented.

The weight of the complete system is 261 g with the ARS-404 and 406 g with the larger ARS-408. The measured weight of the sensors is a bit less in comparison to the specifications. The power supply for the RPi is 5 V. Because of a wide range of the usage and the special use in a UAV, a standard voltage down converter LM2596 on a breakout board was used to power the RPi. This converter allows any power supply from 8 to 40 V DC. Commonly, UAVs have a three-cell battery what is about 12.6 V or more cells e.g., six with 25.2 V. The radar sensors have a wide range of voltage input from 8 to 32 V. Because of this wide range, it is not necessary to load an extra battery. To access the UAV battery, the balancer connector can be applied. Some UAVs provide an extra connector for example to connect the camera and gimbal. If the voltage is in the range it can be used as well. It is worth to highlight that a fuse between the battery and the measurement system is necessary to protect the battery and the UAV as well. A short circuit of the battery can end up with a total crash of the UAV and also in a fire. The common currency of the measurement system is about 0.7 A by 12.7 V. Thereby, a 2 A slow fuse was applied.

**Figure 4:**
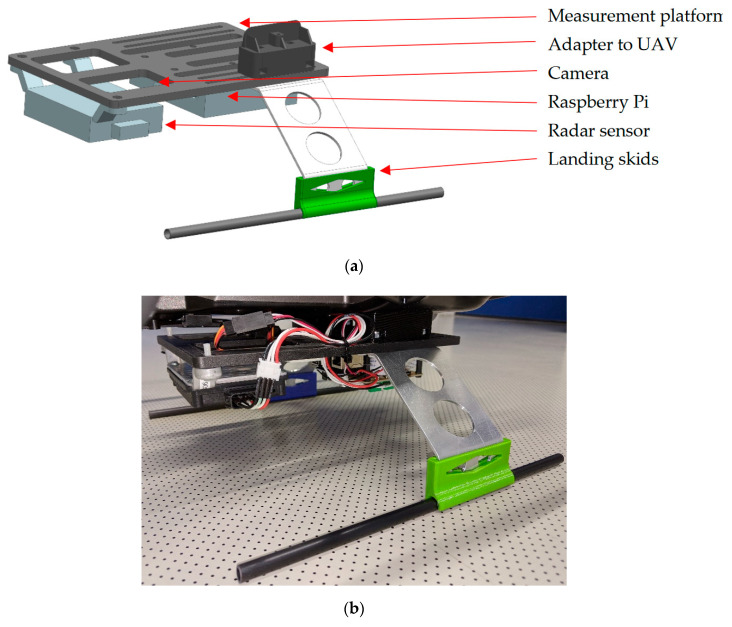
Construction of the bracket system. (**a**) Design of the frame to mount the measurement system and attach it to the unmanned aerial vehicles (UAVs). The adapter to the UAV and the landing skid is only one time shown; the other side is a mirrored copy. (**b**) Attached frame on the UAV.

Besides the measurement system, a bracket is necessary to mount the system on the UAV. It depends on the UAV and is not generally defined. In our case, the bracket is complex because it is a cheap consumer quadcopter Yuneec Q500 and is not build for such a use case. The measurement system is not only suitable for rotary-wing aircraft but also for fixed-wing aircrafts such as [50]. However, to get this UAV ready to carry the measurement system, a bracket was designed. The bracket replaces the landing frame to have enough space for the components, while a minimal weight is needed. The weight is about 200 g and it is 73 g more than the standard landing frame. Figure 4 shows the construction of the bracket.

**Figure 5:**
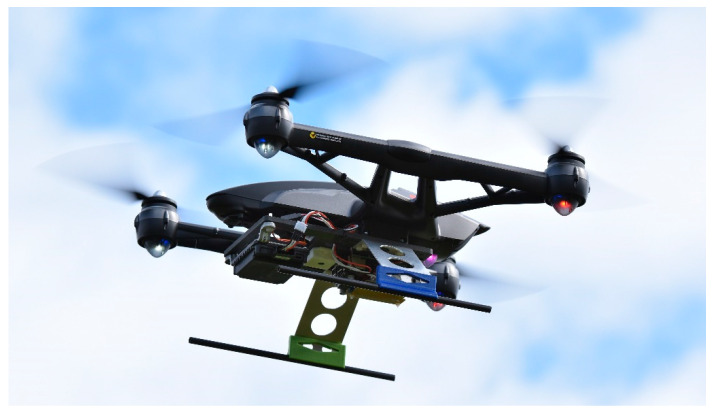
Test flight to check the measurement system with radar sensor ARS-404.

To use the measurement system with another UAV, it is possible to use only the measurement platform and create a new adapter, to fix it on the UAV. The 3D printed measurement platform weights about 54 g and has integrated screw-nuts. Thereby, the radar, camera and RPi can easily mount or replace with screws while the construction is fixed on the UAV. After a successfully mounting of the measurement system and test flights, an assessment of the two sensors was possible. Figure 5 shows the UAV while testing the system with the radar sensor ARS-404. The flight time of the datasheet is “up to 25 min”, whereby a test with camera and gimbal was about 18 min. The same time was reached for the measurement system with ARS-404 and about 10 min for the larger ARS-408. It has to be noted that the flight time greatly depends on weather conditions.

### 2.4. Radar Target

**Figure 6:**
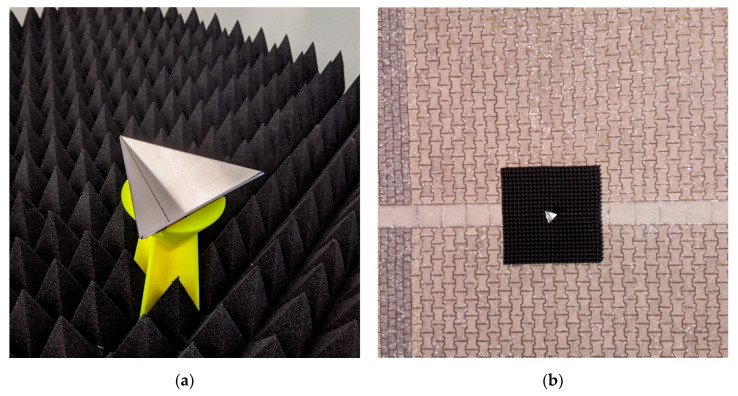
Corner cube reflector with 27.6 m^2^ radar cross-section (RCS) in a 3D printed stand. The reflector is surrounded by absorbing mats in a pyramid style. (**a**) Close-up view from the reflector in the laboratory. (**b**) View from the UAV down to the reflector while test flight.

To verify whether the radar sensor is able to detect targets, we used corner cube reflectors. These are a common method and the result can be assigned to any other materials with a given RCS. The reflector is generally built with three right-angled triangle metal plates. Thereby, two sides have the length “a” and the third side is “a” multiplied by the root of two. The three metal plates assembled with the short side “a” as shown in Figure 6. To calculate the RCS value (*σ*) of the reflector, according to [31] Equation (1) was used:

(1) $$\sigma =\frac{4\pi a^{4}}{3\lambda^{2}}$$

Because of the wide range of the RCS, it is usual to calculate the value in decibel. However, we used reflectors with *a* = 10 and 5 cm. By using the Equation (1) with the wavelength of the radar, the result is *σ*_10_ = 27.6 m^2^ and *σ*_5_ = 1.7 m^2^. In comparison to that, a car has 100 m^2^, a motorcycle 10 m^2^, and a person 1 m^2^ [31]. To get the reflector in the right position, a 3D printed stand was designed. Then, the reflector shows with the open side up to the air. A combination of two or more reflectors is possible for the evaluation. To prevent reflections from the underground, which could disturb the reflector signal, microwave absorbing mats were considered (Pyramid-Absorber C-RAM SFC-4, Emc-Technik & Consulting GmbH, Emilienstraße 35, 70563 Stuttgart, Germany; emc-technik.de). It works in the same way as for acoustic, but the material is different for the high frequency. The design of the absorbing mats is the pyramid style, that is also known from the acoustic [51]. Figure 6 shows a radar reflector with a = 10 cm in a stand, surrounded by absorbing mats.

## 3. Results and Discussion

**Figure 7:**
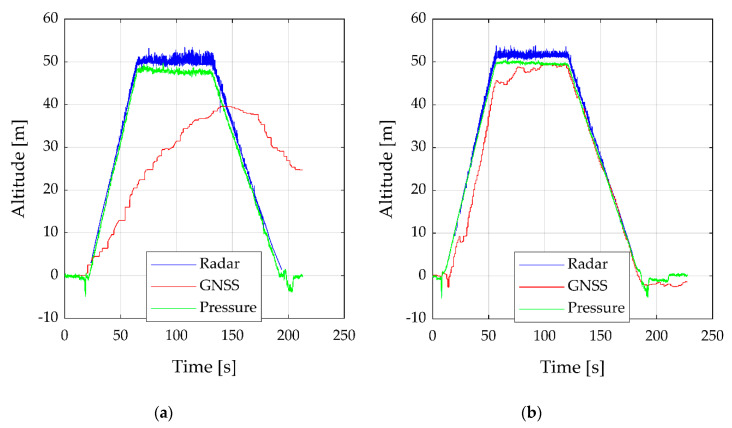
Comparison of the altitude estimation with radar sensor ARS-404, global navigation satellite system (GNSS) sensors, and pressure sensors. The first flight (**a**) shows the flight with the sensor shield GPS-PIE and (**b**) with the OzzMaker shield.

The measurement system is easy to be used and exchange of components such as the radar sensor can be performed without difficulties. Because of the different weights of the radar sensor, after an exchange of the radar, the center of gravity of the UAV has to be verified. The HMI to control the measurement system works stably and the connection via Wi-Fi is robust and wide in the use of different devices. Thereby, all sensors can be valid before conducting a flight. After several tests with the two sensor shields, we decided to use the GPS-PIE Gmm slice. The major reason is because of the better IMU. The BNO055 can directly output the Euler angles and has an automatic calibration. In comparison, the LSM9DS1 has no own calibration and the Euler angles have to be calculated in a post-process. The calibration can be conducted with a special record, the Euler angles can be only calculated if the sample rate is relatively high. Based on the radar sample rate, the used sample rate was too low. Further tests were conducted to check the order of the shields. Thereby, the CAN shield should be the lower and the sensor shield the upper one. This order prevents disturbance from the CAN shield to the IMU. The magnetometer from the BNO055 on the GPS-PIE shield was disturbed from the SPI communication. To prevent this disturbance, it was necessary to cut the SPI0 pins of the connection header, that was used for the communication between the RPi and the CAN shield. This problem could not be solved in another way, even with the manufacturer. A further advantage of this shield position is that the temperature sensor was less impact of the CPU heat. On the other hand, the main disadvantage of the GPS-PIE sensor shield is the lower performance of the GPS. Due to this, two tests were performed to compare the altitude precision of the radar sensor ARS-404 and the GNSS sensors as well as the pressure sensors for both shields. The results are shown in Figure 7.

For this test flight, the UAV ascended to 50 m altitude, stayed there 60 s and descended until touch down. To allow easy comparison, the radar data are filtered, the start value of the GNSS data set to zero and the pressure values converted to meter and set to zero as well.

The radar data in Figure 7a,b have only few clusters lower than 5 m and thereby they are not suitable for short-range applications less than 5 m. The flight altitude, ascent and descent, is well traceable. Because of the side winds, the radar altitude varies while the UAV stays at 50 m altitude. The GNSS sensor on the GPS-PIE shield, shown in Figure 7a, has the worst performance. The start altitude is already about 10 m lower than true. Due to this, the end altitude is higher than the start altitude. Furthermore, the sensor is slow and hangs behind the real altitude. In comparison to this, the GNSS sensor on the OzzMaker sensor shield in Figure 7b follows the altitude except for a few steps. For an application with accurate GPS requirements, we recommend using both sensor shield. This is possible when cutting the UART pins from the header connection, after the lower OzzMaker shield. The pressure sensors show both a better result than the GNSS sensors and therefore, they are appropriate to estimate the flight altitude. The pictures of the attached camera can be used as a reference for the radar data. An example is shown in Figure 6b. Because of the limited quality, the pictures are unsuitable for further processing. For this purpose, a camera with the higher quality should be used in consideration to the weight.

**Figure 8:**
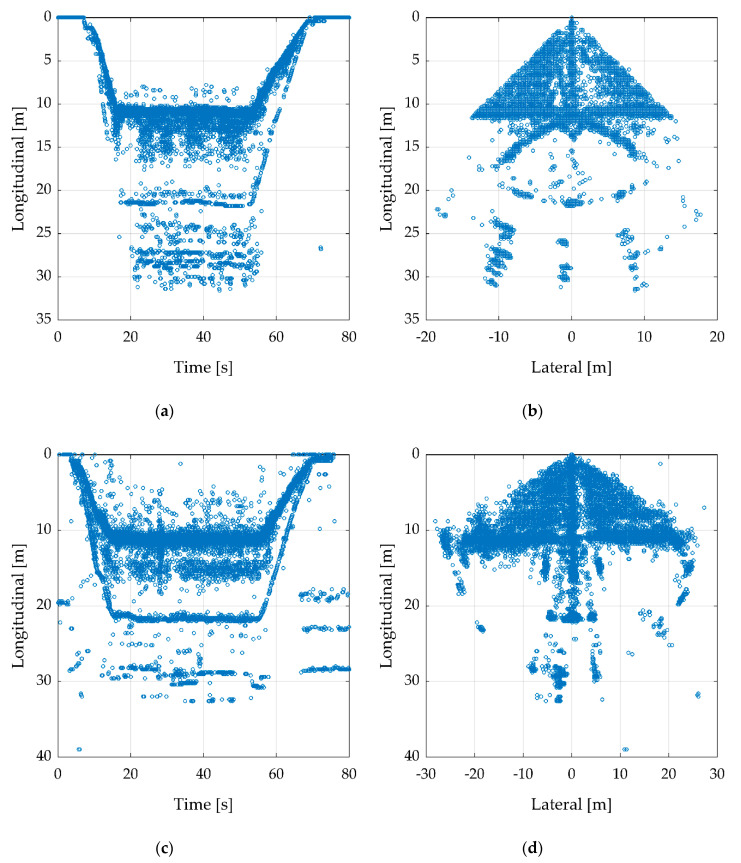
Recording flight in 10 m height over a corner cube reflector. Besides the real target in 10 m distance, several ghost clusters are visible. (**a**,**b**) Flight with ARS-404, to improve the figure the longitudinal axis was cut-off by 35 m and lateral axis by ±20 m. (**c**,**d**) Flight with ARS-408, to improve the figure, the longitudinal axis was cut-off by 40 m and lateral axis by ±30 m.

To compare the two radar sensors, a record was performed with each sensor while the measurement setup is the same. Whereby Figure 8a,b shows the ARS-404 and Figure 8c,d the ARS-408. This example is at 10 m altitude over a reflector surrounded by four absorbing mats as shown in Figure 6b. That view belongs to the radar sensor down to the target, which means, the position of the radar sensor is at longitudinal and latitude zero. The UAV starts from the ground while recording and increase the altitude slowly to 10 m. This can be retraced (Figure 8a) until 15 s as a negative ramp. Then, it stands still over the reflector until 55 s. After that, it touches down and the recording was stopped. The descent can be retraced from 55 to 70 s as a positive ramp and it is slower than the ascent. Even if the altitude is only 10 m, the radar has clusters in a higher distance, e.g., a few clusters between 45 and 50 m. In Figure 8a, these ghost clusters are shown as a line, e.g., by about 20 m.

Figure 8b shows several ghost clusters on a circular path with separate hot spots. The same measurement was recorded with the ARS-408. In comparison to the smaller ARS-404, the ARS-408 has more clusters with a higher RCS value due to his larger size and thereby larger antennas. The flight path could be retraced, just like the ARS-404, the ascent and descent are shown as ramps. This can be considered different as the ARS-408 has more ghost clusters between the target and the radar sensor (Figure 8c). Further, the circular path looks different in Figure 8d. The ghost clusters are a product of several radar effects such as multipath receiving or scattering of the transmitted signal. The surrounding can have a negative influence if there are objects such as parking light masts, manhole covers, or cars that can reflect the signal with a high RCS value. Further causes are discussed in the recent radar literature by several authors [18,19,20,21].

**Figure 9:**
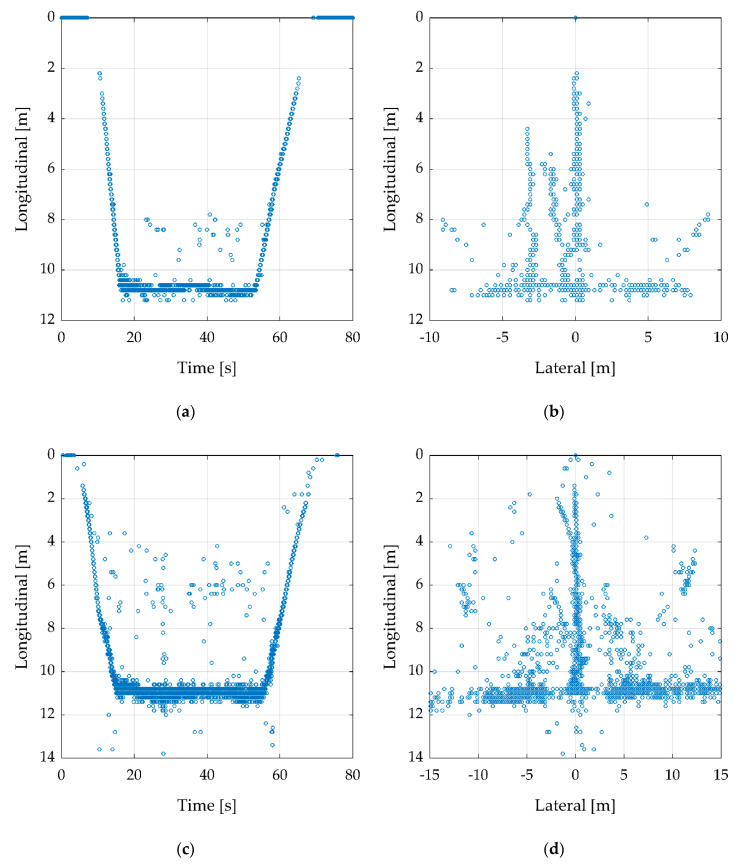
Based on Figure 8, this figure is filtered with min- and max-filters by the values shown in Table 4. (**a**,**b**) with the radar sensor ARS-404, (**c**,**d**) with ARS-408.

To improve the data, a filter tool was used. Figure 9 shows the result obtained from Figure 8 after applying min- and max-filters. The values and specifications are shown in Table 4.

After the min- and max-filters, the results are clearer to be interpreted. In Figure 9a,c, the ramps, as well as the stand still of the UAV, can be recognized. There are still several single ghost clusters. Because they do not build hot spots, further filtering should not find any issue. In Figure 9b,d, the clusters look like a turned T. Thereby, the vertical line is the ascent and descent ramps and the horizontal line the underground, while the UAV stands still at about 10 m altitude.

**Figure 10:**
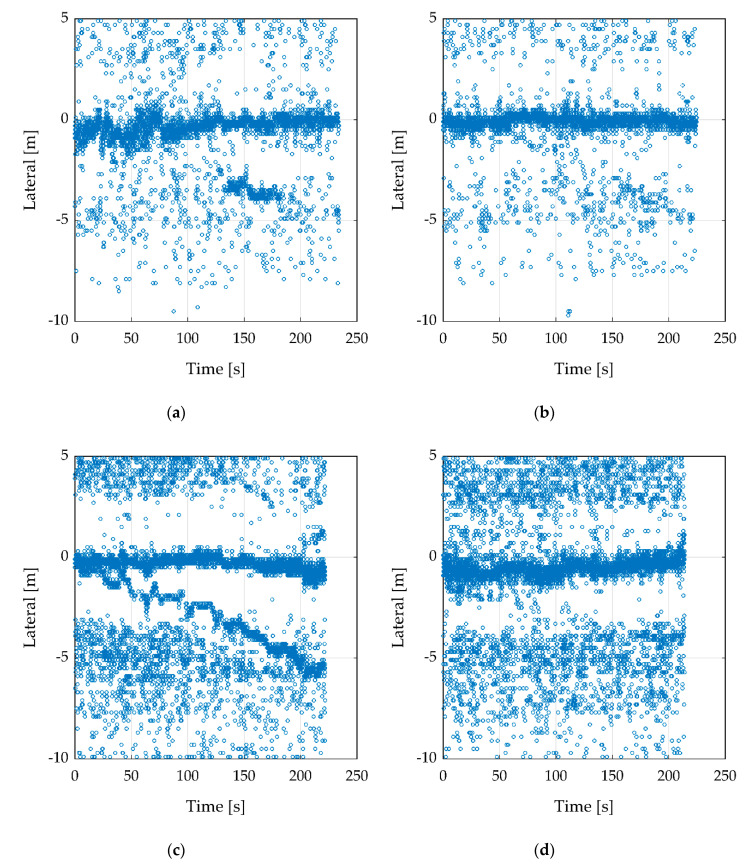
Recording flight in 10 m height over two corner cube reflectors. One reflector position is fixed, the second reflector was pulled away by 50 cm steps. (**a**,**b**) with the radar sensor ARS-404, (**c**,**d**) with ARS-408. The RCS of the reflector is in (**a**,**c**) 27.6 m^2^ and (**b**,**d**) 1.7 m^2^.

The next test was carried out using two corner reflectors. Thereby, one reflector was fixed on the position and the second one was pulled away by 50 cm steps each 20 s on the lateral axis to the right side (lateral minus). The measurement starts with a distance of about 50 cm and ends by 4 m. The flight altitude was 10 m and the UAV stood steady above the first fix reflector. This measurement was performed for both radar sensors (Figure 10). To improve the figure, the clusters were filtered by the values presented in Table 5.

Figure 10a shows the ARS-404 with the large reflector. The fixed reflector can be seen on the lateral axis by 0 m as a line of clusters. The second reflector, that is pulled away, is only clearly visible as a hot spot around 150 s. In the second figure (Figure 10b), the same measurement was conducted with the small reflector. In comparison to the large reflector, there is no clear hotspot. With the knowledge of the true path, the path of the second reflector can be obtained but is not visible.

Figure 10c presents the best results with the ARS-408 and the large reflector. Thereby, the path of the second reflector is well traceable. In Figure 10d the small reflector was used and the results are similar to Figure 10b. The path is not traceable.

To extract the features of a record, in this research a corner cube reflector, an advanced filter mechanism is necessary. The presented examples are only filtered with min- and max-filters. Due to more complex features, it is not possible to detect them with such a filter tool. A feature extraction such as gullies and rills are conceivable. The main reason is the reliable distance measurement of the radar. Also, it provides information about soil conditions. The rough ground will result in a low RCS value, a flat ground in a higher RCS. The evaluation of the gullies or rills can be compared to a ground reference point (e.g., corner cube reflector), GNSS point or with the same measurement in time intervals. This can be similar to other ground topographical measurements used with photogrammetric techniques to estimate accurate cross-sectional measurements which are less expensive and time-demanding [52,53]. To forecast a landslide or flash flood, larger areas has to be considered as was mentioned by recent investigations including sensors and UAVs [54,55]. This requires a fixed-wing aircraft with a larger range than a rotary-wing aircraft. The detection of ground information can be disturbed by plants and thereby the number of useable clusters of the ground decreases. Nevertheless, the results can improve the forecast, because of additional information about the ground. River evolution paying attention to the meander changes after land-use changes or rainfall events can be monitored temporally by the radar sensor as other authors also confirmed [56]. Further, after occurring special events such as heavy rainfalls and the consequences on the surface can be monitored by this radar like [57,58]. Similar methods can be used to monitor aeolian processes [59] and impacts of land-use changes, e.g., in vineyards [60]. Besides specific research fields, it is possible to combine the radar sensor information with optical data. Thus, it is possible to add a third dimension to a two-dimension picture.

## 4. Conclusions

This paper presents a new advance to use a high-performance automotive technology for potential earth science research. Thereby, the gap between professional and low-cost radar sensors could be reduced. To use this system, no deep radar knowledge is necessary and pre-/post-events assessments can be done. The radar sensors ARS-404 and ARS-408 from Continental were introduced with the basic radar techniques and the most important specifications. To use such a sensor, a measurement system was presented to record the radar data. The results of a recording are saved into a file and can be shown as a point cloud. The system was extended with sensors such as GNSS, IMU, temperature, and pressure. The whole system weighs about 315 g with the smaller ARS-404 and 450 g with the ARS-408. Thereby, the results confirmed good insights into how the radar data looks like. To use the system for a specific application, further work is necessary, specifically for some applications, a 3D data analysis would be relevant. Because the radar sensor measures in 2D, the flight path can be used to get the third dimension. These records would need special data post-processing. Further, due to the flood of data, suitable filters are necessary to extract the desired data. To extract more complex targets, the development of a filter chain would be necessary. Despite the upcoming challenges, a further investigation is worthwhile, because the development in the automotive sector is ongoing and will bring new high-performance radar sensors to the market.

## Figures and Tables

**Table 1:** Specifications of the ARS-404 and ARS-408 radar sensors [49].

Specification	ARS-404	ARS-408
Voltage	+8.0 … 32 V DC	+8.0 … 32 V DC
Current	375 mA by 12 V	550 mA by 12 V
Power consumption	4.5 W	6.6 W
Weight	172 g	320 g
Size	136 × 68 × 34 mm	137 × 91 × 31 mm
Interface	High-Speed CAN	
Refresh rate	50 ms	60 ms
Range far/near area	170 m/70 m	250 m/70 m
Resolution far/near area	0.4 m/0.4 m	1.79 m/0.39 m
Beam horizontal far/near area	±9°/±45°	±9°/±60°
Resolution horizontal far/near area	3.3°/6.6°	1.6°/3.2°
Beam vertical far/near area	±18°/±18°	±14°/±20°
Frequency	76–77 GHz	
Wavelength	3.94–3.89 mm	
Cost (approx.)	€2500	

**Table 2:** Sensor types of the two tested shields.

Name	GPS-IMU v3	GPS-PIE Gmm Slice
GNSS	uBlox CAM-M8	GlobalTop Gmm-u1
IMU	STMicroelectronics LSM9DS1	Bosch BNO055
Pressure and Temperature	Bosch BMP280/388	TE Connectivity MS5637
Manufacturer	OzzMaker, PO Box Q326, Queen Victoria Building, NSW 1230 Australia; ozzmaker.com	The BlackBoxCamera, Office 102, 61 Willow Walk, Tower Bridge, London SE1 5SF, United Kingdom; gps-pie.com
Cost	AUD$62.00	£22.99

**Table 3:** Weight of the components of the measurement system.

Name	Type	Weight
Raspberry Pi with shields	3B	106 g
Raspberry Camera	Camera V2	4 g
Radar sensor	ARS-404	151 g
Radar sensor	ARS-408	296 g

**Table 4:** Filter setup for the min- and max-filter to improve Figure 8.

Specification	Min	Max
Longitudinal	-	20 m
Lateral	-15 m	15 m
RCS	0 m^2^	-

**Table 5:** Filter setup for Figure 10 with min and max values.

Specification	Min	Max
Longitudinal		20 m
Lateral	−10 m	5 m
RCS	0 m^2^	
